# Effect of a Roughness Element on the Hypersonic Boundary Layer Receptivity Due to Different Types of Free-Stream Disturbance with a Single Frequency

**DOI:** 10.3390/e21030255

**Published:** 2019-03-06

**Authors:** Mingfang Shi, Lidan Xu, Zhenqing Wang, Hongqing Lv

**Affiliations:** 1 College of Aerospace and Civil Engineering, Harbin Engineering University, Harbin 150001, China; 2 School of Civil Engineering, Inner Mongolia University of Science and Technology, Baotou 014010, China

**Keywords:** freestream disturbances, FFT, hypersonic boundary layer, receptivity

## Abstract

The hypersonic flow field around a blunt cone was simulated using a high-order finite difference method. Fast acoustic waves, slow acoustic waves, entropy waves, and vortical waves were introduced into the free-stream to determine the influence of a free-stream with disturbances on the hypersonic flow field and boundary layer. The effect of disturbance type on the evolution of perturbations in the hypersonic boundary layer was analyzed. Fast Fourier Transform was adopted to analyze the effect of the disturbance type on the evolution of different modes in the boundary layer. A roughness element was introduced into the flow field to reveal the impact of the roughness element on hypersonic boundary layer receptivity. The results showed that a free-stream with disturbances affected the hypersonic flow field and boundary layer; acoustic waves had the greatest influence. The impact of slow acoustic waves on the flow field was mainly concentrated in the region between the shock and the boundary layer, whereas the influence of fast acoustic waves was mainly concentrated in the boundary layer. Multi-mode perturbations formed in the boundary layer were caused by the free-stream with disturbances, wherein the fundamental mode was the dominant mode of the perturbations in the boundary layer caused by fast acoustic waves, entropy waves, and vortical waves. The dominant modes of the perturbations in the boundary layer caused by slow acoustic waves were both the fundamental mode and the second harmonic mode. The roughness element changed the propagation process of different modes of perturbations in the boundary layer. In the downstream region of the roughness element, perturbations in the boundary layer caused by the slow acoustic waves had the greatest influence. The second harmonic mode in the boundary layer was significantly suppressed, and the fundamental mode became the dominant mode. The effects of fast acoustic waves and entropy waves on the boundary layer receptivity were similar, except the amplitude of the perturbations in the boundary layer caused by the fast acoustic waves was larger.

## 1. Introduction

The transition of the hypersonic boundary layer has a considerable impact on the control and propulsion performance of a hypersonic vehicle and placing higher demands on the thermal protection system (TPS) of a hypersonic vehicle [1,2,3]. Therefore, investigating the transition mechanism of the hypersonic boundary layer is crucial. Compared with the incompressible flow and supersonic flow, the flow state of the hypersonic boundary layer is complex. Morkovin [4] stated that the transition of the hypersonic boundary layer can be divided into several paths according to the level of the disturbances in the free-stream. The boundary layer transition must go through the receptive phase under any level of disturbance. The hypersonic boundary layer receptivity refers to the response of the hypersonic boundary layer to environmental disturbances. For example, hypersonic boundary layer receptivity under free-stream disturbance refers to the process that occurs when disturbances in the free-stream enter the boundary layer and result in the generation of perturbations in the boundary layer. Many scholars have researched hypersonic boundary layer receptivity [5,6,7,8,9,10,11,12,13,14], but it is not yet fully understood because the hypersonic boundary layer receptivity is affected by many factors. These factors interact with each other, which increases the complexity of the hypersonic boundary layer receptivity.

The factors that impact transition are divided into two categories: free-stream disturbances and wall disturbances [5]. Free-stream considerably affects the boundary layer because the parameters of free-stream, such as velocity (or Mach number), temperature, and Reynolds number, can change the hypersonic boundary layer receptivity. The hypersonic boundary layer receptivity becomes more complex when disturbances are added to the free-stream. The type of free-stream disturbances significantly impacts the hypersonic boundary layer receptivity, and free-stream disturbances include many kinds, such as acoustic waves (including fast and slow acoustic waves), vortical waves, and entropy waves [6]. Cerminara [7] analyzed the leading edge receptivity of supersonic and hypersonic flow over a blunt wedge under acoustic waves by direct numerical simulation and found that fast acoustic waves lead to drag and reflection waves with high amplitude in the hypersonic flow field, whereas slow acoustic waves lead to low amplitude convected waves. The relationship between wall pressure fluctuations and free-stream disturbances was also revealed. Duan et al. [8] analyzed the spatial evolution of pressure fluctuations in the turbulent boundary layer at Mach 5.86 by direct numerical simulation. They found that the dominant mode of the acoustic waves in free-stream occurs in a lower frequency region, a larger spatial extent, and a smaller bulk propagation speed. Jagadeesh et al. [9] used the Schlieren imaging technique to measure the unstable motion of acoustic waves in the boundary layer and reported that this method could be used to study laminar-turbulent transition and the flow in a large gradient region. Compared with acoustic waves, the influence of other types of free-stream disturbance on hypersonic boundary layer is less studied. Qin and Wu [10] discussed the effects of acoustic waves, entropy waves, and vortical waves in a free-stream on the hypersonic boundary layer receptivity over a blunt wedge. They found that shock changed the evolution of the perturbations in the boundary layer under three kinds of free-stream disturbances. They also reported that these three kinds of free-stream disturbances could generate unsteady perturbations in the boundary layer without considering the interaction between the free-stream disturbances or the roughness element on the wall. Miselis et al. [11] combined the direct numerical simulation method and the multiple mode decomposition method to examine the hypersonic boundary layer receptivity under entropy waves in free-stream. The results of the numerical simulation were in good agreement with the experimental results. The parameters of free-stream disturbances, such as amplitude, frequency, and incident angle, also significantly impact hypersonic boundary layer receptivity. Balakumar et al. [12] analyzed the receptivity of the supersonic boundary layer under acoustic waves. They found that the perturbations in the boundary layer are a response to the free-stream acoustic waves, and the amplitude of the perturbations in the boundary layer is related to the incident angle of the free-stream disturbances. Tang et al. [13,14] simulated the hypersonic flow field under different amplitudes and frequencies of free-stream disturbances. They found that the amplitude and frequency of the free-stream disturbances change the transformation process of the dominant mode in the boundary layer.

The second kind of factor influencing hypersonic boundary layer receptivity is wall disturbances, including blow-suction disturbances [15], wall deformation [16], local heating/cooling on the wall [17], and nose-tip bluntness [18,19]. The effect of roughness elements on the hypersonic boundary layer receptivity mechanism has been researched. The influence of roughness element shape, location, height, and number [20,21,22,23] on the receptivity and stability mechanism of hypersonic boundary layer have been widely studied. The receptivity mechanism of free-stream disturbances in the hypersonic boundary layer is, to some extent, changed by the roughness element. We studied the impact of roughness element height on the hypersonic boundary layer receptivity under pulse entropy waves. We found that the increase in the roughness element height causes the position of the dominant mode in the hypersonic boundary layer to move upstream [24]. Zhao et al. [25] combined an experimental method and numerical simulation to analyze the impact of roughness element height on the supersonic boundary layer receptivity. The results showed that the height of an isolated roughness element affected the path of transition. When the roughness element is low, the transition is dominated by shear layer instability; when the roughness element is high, the strong instability of the separation region leads to boundary layer transition. Duan and Zhong [26,27,28] introduced a new high-order finite difference method called the high-order cut-cell method. The influence of the height and position of the roughness element on the receptivity of the hypersonic boundary layer under free-stream disturbances can be analyzed using this method. David et al. [29] presented an input-output analysis method to study the impact of roughness elements on the transition of the boundary layer.

The purpose of this research was to study the effect of the type of single-frequency free-stream disturbances on the hypersonic boundary layer receptivity with a roughness element. A high-precision method was adopted to numerically simulate the hypersonic unsteady flow field, and the formation and the evolution of the perturbations in hypersonic boundary layer were determined. The response mechanisms of the hypersonic flow field and the boundary layer to different types of free-stream disturbance with a smooth wall were analyzed first. Then, the evolution process of wall pressure disturbance in the time and space domains was analyzed. Fast Fourier Transform (FFT) was adopted to convert the disturbance signals in the time domain into the frequency domain. The evolution of the perturbations in different modes in the boundary layer was obtained and the receptivity mechanism of the hypersonic boundary layer was revealed. Finally, the roughness element was introduced to examine the influence of the roughness element on the propagation process of different modes of perturbation in the hypersonic boundary layer under different types of free-stream disturbance.

## 2. Governing Equations and Numerical Methods

To accurately simulate the hypersonic flow field with a roughness element under the free-stream disturbances, we adopted the high-order finite difference method to simulate the hypersonic flow field. The generation and evolution of perturbations in the hypersonic boundary layer were analyzed to reveal the mechanism of the hypersonic boundary layer receptivity with a roughness element under free-stream disturbances. The equations governing the numerical simulation are the two-dimensional conservation Navier–Stokes equations in a Cartesian coordinate system. For convenience, the governing equations are converted from a Cartesian coordinate system to a curvilinear coordinate system. The expression is as follows:

(1) $$\frac{\partial U}{\partial t}+\frac{\partial F_{i}}{\partial x_{i}}+\frac{\partial F_{\upsilon i}}{\partial x_{i}}=0\;\left(i=1,2\right)$$

where *U* is a variable vector and ***F****_i_* and ***F****_υi_* are inviscid flux and viscous flux in the *i*th spatial direction, respectively. They are expressed as:

(2) $$\begin{array}{l} U={\left[\rho ,\rho u_{1},\rho u_{2},\rho e\right]}^{T},\\ F_{i}={\left[\rho u_{i},\rho u_{1}u_{i}+p\delta_{1}{}_{i},\rho u_{2}u_{i}+p\delta_{2}{}_{i},(\rho e+p)u_{i}\right]}^{T},\\ F_{\upsilon i}={\left[0,\tau_{1}{}_{i},\tau_{2}{}_{i},\tau_{ij}-q_{i}\right]}^{T} \end{array}$$

where ρ, *u*, and *p* refer to the density, velocity, and pressure of hypersonic flow field, respectively; *δ*, *e,* and *τ* are the Kronecker symbol, total energy, and shear stress, respectively. Only the hypersonic flow consistent with the hypothesis of perfect gas was considered, and

(3) p=ρRT

(4) $$e=\rho C_{v}T+1/2u_{j}u_{j}$$

(5) $$\tau_{ij}=\mu \left(\frac{\partial u_{i}}{\partial x_{j}}+\frac{\partial u_{j}}{\partial x_{i}}\right)+\delta_{ij}\lambda \frac{\partial u_{k}}{\partial x_{k}}$$

(6) $$q_{i}=-k\frac{\partial T}{\partial x_{i}}$$

where *R* and *C_v_* are the gas constant and specific heat, respectively; *k* and *µ* are the heat conductivity coefficient and viscosity coefficient, respectively.

Numerical simulation of a hypersonic flow field under complex conditions requires the use of a high-order finite difference method given the many tiny flow structures, large gradient regions, and discontinuous regions in the hypersonic flow field [30,31,32,33]. There are many high-order finite difference methods, among which the central difference scheme applies a relatively simple logic relationship, is easy to program, and requires less computation. Therefore, this method is widely used for the numerical simulation of hypersonic flow fields. This scheme may introduce serious numerical oscillation in discontinuous regions and large gradient regions. In order to address this problem, the flux splitting scheme is introduced into the numerical simulation. This scheme does not generate numerical oscillation when capturing shocks. It has high reliability and is relatively simple. However, this method can lead to large numerical dissipation, which increases the error in the viscous region. The weighted essentially non-oscillation scheme (WENO) can effectively solve this problem. The WENO scheme was developed on the basis of the essentially non-oscillatory scheme. This method is highly precise and provides high resolution in the process of solving conservation equations and can obtain results without numerical oscillation. Therefore, this high-precision method was used to simulate the hypersonic flow field. The Steger-Warming flux splitting method was adopted to split the inviscid term into positive and negative flux terms. Then the fifth-order WENO scheme was used to solve them. The viscous term was solved using a six-order central difference scheme. Finally, the three-step and three-step total variation diminishing Runge-Kutta schemes were adopted in the time terms. Because the method has high precision and resolution, and there is no fluctuation when capturing shock, this method is widely adopted in the numerical simulation of hypersonic flow fields. The verification of numerical methods and codes has been provided in detail in previous articles and is not outlined here [26,27,34].

## 3. Calculation Model and Conditions

**Figure 1:**
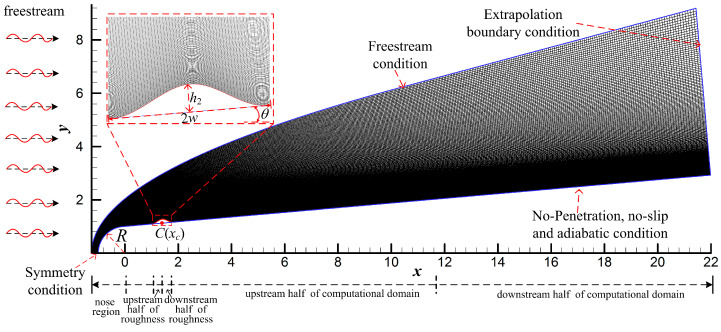
Computational model and grid.

In this study, we analyzed the hypersonic boundary layer receptivity with a roughness element under free-stream disturbances. The calculation model was the hypersonic flow field around a blunt cone under free-stream disturbances, as shown in Figure 1. The inlet boundary of the computational domain is the free-stream condition, and the exit condition is the extrapolated condition. The symmetric condition is adopted at *y* = 0, and non-penetrating and non-slip adiabatic conditions are adopted on the wall. The free-stream parameters in the paper are shown in Table 1, where, *Re*, and *Ma* represent the angle of attack, Reynolds number, and Mach number, respectively. The subscript ∞ represents a free-stream parameter. The variables are all dimensionless. The dimensionless variables were obtained by dividing the instantaneous variables of the flow field by the corresponding freestream variables.

To determine the influence of the free-stream disturbance type on the generation and evolution of perturbations in the hypersonic boundary layer, we first numerically simulated the steady flow field, and then the free-stream disturbances were superimposed on the upper boundary of the computational domain. The moment of flow field becoming steady was defined *t* = 0 and then free-stream disturbances were added into the flow field until the end of the calculation. This kind of free-stream disturbance is called a continuous wave. Free-stream disturbances include four kinds of disturbances: fast acoustic waves, slow acoustic waves, entropy waves, and vortical waves. Their expressions are as follows [35]:

(7) $$\begin{array}{ll} \mathrm{Fast}\;\mathrm{acoustic}\;\mathrm{wave}:\; & \mathrm{Slow}\;\mathrm{acoustic}\;\mathrm{wave}:\\ \left[\begin{array}{l} u^{\prime }\\ v^{\prime }\\ p^{\prime }\\ \rho^{\prime } \end{array}\right]=\left[\begin{array}{l} \varepsilon \\ 0\\ \varepsilon /Ma\\ \varepsilon Ma \end{array}\right]e^{i(kx-\frac{f\cdot Re}{10^{6}}t+\frac{\pi }{2})}\; & \left[\begin{array}{l} u^{\prime }\\ v^{\prime }\\ p^{\prime }\\ \rho^{\prime } \end{array}\right]=\left[\begin{array}{l} \varepsilon \\ 0\\ -\varepsilon /Ma\\ -\varepsilon Ma \end{array}\right]e^{i(kx-\frac{f\cdot Re}{10^{6}}t+\frac{\pi }{2})}\\ \mathrm{Entropy}\;\mathrm{wave}:\; & \mathrm{Vortical}\;\mathrm{wave}:\\ \left[\begin{array}{l} u^{\prime }\\ v^{\prime }\\ p^{\prime }\\ \rho^{\prime } \end{array}\right]=\left[\begin{array}{l} 0\\ 0\\ 0\\ \varepsilon Ma \end{array}\right]e^{i(kx-\frac{f\cdot Re}{10^{6}}t+\frac{\pi }{2})}\; & \left[\begin{array}{l} u^{\prime }\\ v^{\prime }\\ p^{\prime }\\ \rho^{\prime } \end{array}\right]=\left[\begin{array}{l} 0\\ \varepsilon \\ 0\\ 0 \end{array}\right]e^{i(kx-\frac{f\cdot Re}{10^{6}}t+\frac{\pi }{2})} \end{array}$$

where, *k*, and *f* represent the amplitude *ε* = 6 × 10^–2^, number of waves *k* = 3.1446 × 10^–4^, and frequency of the free-stream disturbance *f* = 50π, respectively. Therefore, the period of free-stream disturbances is four. The superscript ′ represents the fluctuation quantity, and the instantaneous fluctuation quantity in the flow field can be obtained by subtracting the value of the instantaneous variable in the unsteady flow field from the value of steady flow field. To simplify the description without affecting the distinction, “fast acoustic waves” and “slow acoustic waves” are referred to as "fast waves" and “slow waves” hereinafter, respectively.

In this paper, the roughness element is controlled by a cubic polynomial, as follows:

(8) $$y=\frac{R_{n}+h}{\cos\theta }+x\tan\theta -\frac{3h}{\cos\theta }{\left(\frac{x-x_{c}+h\sin\theta }{h\sin\theta \pm w\cos\theta }\right)}^{2}+\frac{2h}{\cos\theta }{\left(\frac{x-x_{c}+h\sin\theta }{h\sin\theta \pm w\cos\theta }\right)}^{3}$$

where *R*, *θ*, *h*, *w*, and *x_c_* respectively represent the radius of the blunt cone nose, half cone angle, the height of the roughness element, half-width, and center coordinates, as shown in Table 2. The height of the roughness element was selected according to the thickness of the local boundary layer. Under the calculation conditions in this paper, the thickness of the boundary layer in the steady flow field is 0.1992, so the height of roughness element was selected as 0.15, which is 75% of the thickness of the local boundary layer. The computational grids were 801 × 251, and the grids were clustered in the region near the wall and nose using the exponential stretching method because the gradient of flow parameters in this area is large.

## 4. Effect of Free-Stream Disturbances on Hypersonic Flow Field and Boundary Layer

**Figure 2:**
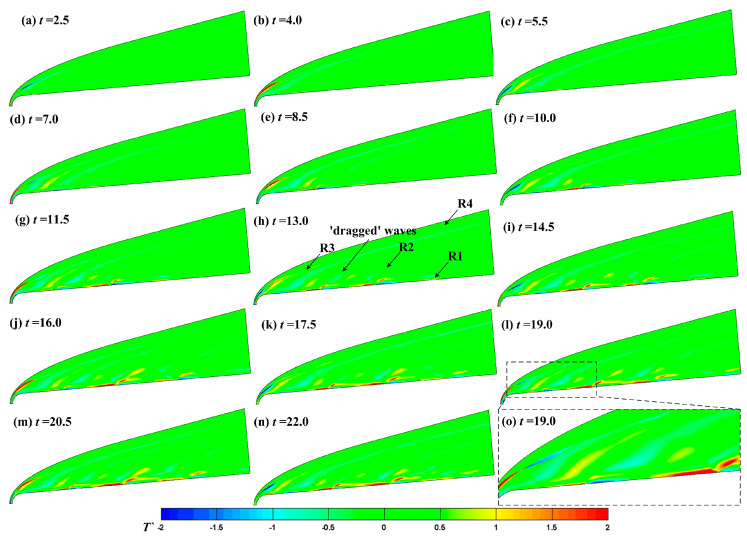
Contours of transient temperature fluctuation in a flow field at different times.

We wanted to accurately analyze the influence of roughness elements on hypersonic boundary layer receptivity under different types of disturbance. Firstly, the hypersonic flow field on the smooth wall was numerically simulated to determine the formation and evolution of disturbances in the hypersonic flow field and boundary layer under different types of disturbance. The effect of fast waves on the hypersonic flow field and boundary layer was used as an example to observe the response process of the hypersonic flow field to free-stream disturbances. Figure 2 shows the evolution of the temperature fluctuations in a hypersonic flow field under fast waves. Again, the physical quantities in this paper are dimensionless. Figure 2 shows that the fast waves pass through the shock and disturbances form and evolve in the flow field, boundary layer, and downstream. When *t* = 15, the free-stream disturbances totally passed through the flow field, so when *t* > 15, the response of the hypersonic flow field and boundary layer to free-stream disturbances is periodic. The unsteady hypersonic flow field can be simply divided into four regions as shown in Figure 2h. The first region is the boundary layer region, which is labelled region R1. The fast waves enter the boundary layer after passing through the shock and excite perturbations in the boundary layer. The perturbations result in a high-temperature spot and a low-temperature spot in the boundary layer during the process of propagating downstream. The R2 region is between the boundary layer and the shock. The fast waves enter the flow field through the shock and dragged waves form, as shown in Figure 2h. The amplitude of the disturbances in this region is obviously lower than that in the boundary layer. The disturbances in the flow field interact with the perturbations in the boundary layer, and the disturbances at the boundary layer edge no longer present a high-temperature spot and a low-temperature spot in sequence. The R3 region is the shock region. The shock deforms obviously, and the amplitude of the disturbances changes most obviously with the normal shock. With the decrease of shock intensity, the amplitude of the disturbances decreases significantly. The R4 region is the outer region of the shock. Fast waves enter the flow field from the upper boundary of the computational domain until they interact with the shock.

**Figure 3:**
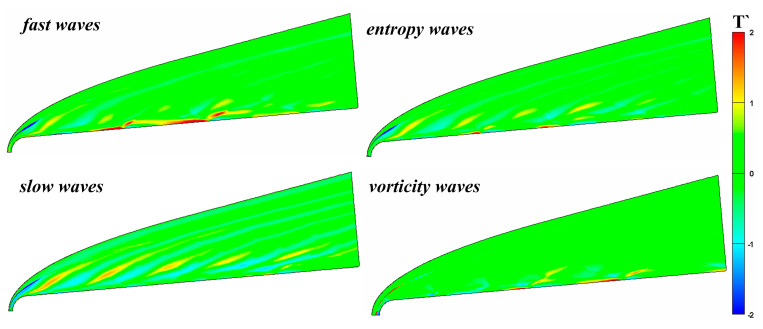
Contour of transient temperature fluctuation under different type free-stream disturbances.

Figure 3 compares the instantaneous temperature field under continuous free-stream disturbances, including fast and slow waves, vortical waves, and entropy waves. The hypersonic flow field and the boundary layer considerably differ in response to different types of free-stream disturbance. The regions most influenced by the disturbance type in the hypersonic flow are regions R1 and R2: the boundary layer and the region between the boundary layer and shock wave. After passing through the shock wave, the slow waves result in a series of regular “drag” waves in R2. Although the other three disturbance waves form a series of drag waves, their intensities are less than that of the slow wave, a little drag wave even occurs under vortical waves. Cerminara et al. [7] conduct a numerical simulation of hypersonic flow field under the acoustic waves and their results are very similar to those in this paper. However, the amplitude of the acoustic waves (1 × 10^−4^) was small in their simulation, so the bow shock does not undergo significant deformation. However, in the boundary layer (R2), slow waves do not form a significant temperature spot structure in the boundary layer, whereas fast waves, entropy waves, and vortical waves all form significant high-temperature spots and low-temperature spots in the boundary layer. Of these, the change caused by fast waves is the most significant. In other words, the acoustic waves most influence the flow field, followed by the entropy waves, and the vortical waves have the least influence. The influence of slow waves on the flow field is mainly concentrated in the region between the shock and the boundary layer, whereas the influence of fast waves is mainly concentrated in the boundary layer. From the perspective of hypersonic boundary layer receptivity, fast waves, entropy waves, and vortical waves similarly influence the hypersonic boundary layer, whereas the influence of slow waves is quite different from the other three disturbances. Kara [36] stated that this occurs because the fast waves resonate with the boundary layer in the nose region of the blunt cone, amplifying the perturbations in the boundary layer. The resonance of the fast waves causes the formation of entropy waves and vortical waves, so the propagation processes of the three in the boundary layer are similar.

**Figure 4:**
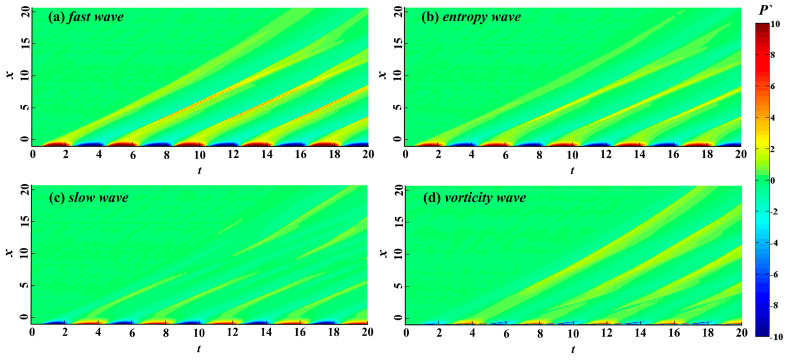
Space-time pressure fluctuation contour map under the smooth wall.

The free-stream disturbances significantly change the flow structure of the hypersonic flow field and also destroy the flow structure and temperature distribution in the boundary layer. However, the perturbations with different modes in the boundary layer have different receptivity to temperature. Therefore, the free-stream disturbances change the mechanism of hypersonic boundary layer receptivity and even change the flow state in the boundary layer. Figure 4 illustrates the evolution of wall pressure fluctuation in the time and space domains under different types of free-stream disturbances, which we used to analyze the influence of free-stream disturbances on the evolution of perturbations in the boundary layer. In Figure 4, the continuous free-stream disturbances cause the hypersonic flow field to alternately form a significant high-pressure region and a low-pressure region in the time domain in the nose region. The free-stream disturbances are significantly enlarged in the nose region and rapidly decrease at the edge of the nose region. In the nose region, the perturbations in the boundary layer caused by fast waves are the strongest, whereas the perturbations in the boundary layer caused by vortical waves are the weakest and no longer present regular sinusoidal distribution. Notably, in the downstream region of the nose region, there is no obvious attenuation stage in the evolution process of the perturbations caused by vortical waves in the boundary layer.

**Figure 5:**
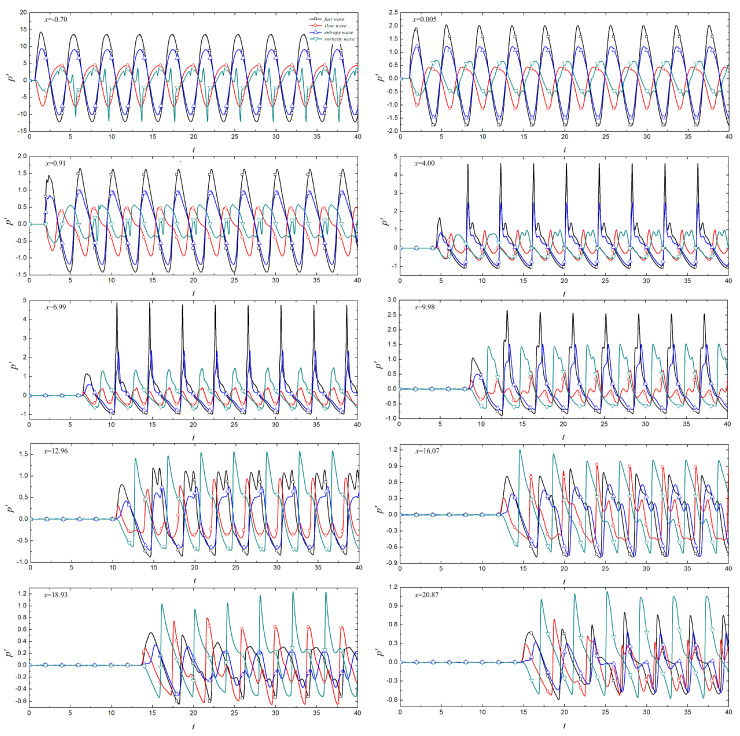
Time history trace of wall pressure fluctuation at various streamwise locations under different types of free-stream disturbance.

To better compare the difference in the propagation of the perturbations in the boundary layer caused by different types of free-stream disturbance in the time domain, Figure 5 shows the propagation process of the pressure fluctuation at different positions in the boundary layer in the time domain under free-stream disturbances. The boundary layer experiences free-stream disturbances later in the more downstream region. In the nose region, the waveform of the perturbations is closer to free-stream, and the amplitude of the perturbations in this region is significantly larger than that in the downstream region, even an order of magnitude higher than the perturbations at some locations in the downstream region. From the nose region to *x* = 9.98, the amplitude of the perturbations in the boundary layer caused by fast waves is the largest. In the region of *x* = 9.98–20.87, the amplitude of the perturbations in the boundary layer caused by vortical waves are the largest. In the time domain, the response process of the boundary layer to free-stream disturbances can be divided into two stages. In the first stage, free-stream disturbances interact with the boundary layer, resulting in the generation of perturbations in the boundary layer. This is called the response phase. There are some differences in the response time of different types of disturbances at different positions in the response phase. The second stage involves periodic pressure fluctuations in the boundary layer, which is called the periodic phase. The magnitude relationship between the amplitude of the perturbations in the response phase and in the periodic phase is affected by the type of the disturbances and their locations. At *x* = 4.00, the amplitude of the perturbations in the boundary layer caused by fast waves is significantly smaller in the response phase than in the periodic phase, and this phenomenon remains until *x* = 12.96. For fast waves, the amplitude of the perturbations in the boundary layer in the response phase is smaller than that in the periodic phase, except at *x* = 18.937. The amplitude of the perturbations in the boundary layer caused by the entropy waves may be larger than the amplitude of the periodic phase, such as *x* = 16.07 and *x* = 20.87; the amplitude may be smaller than in the periodic phase, such as *x* = 12.967.

The waveform of the perturbations in the boundary layer caused by different types of free-stream disturbance becomes more complicated as the perturbations propagate downstream, and the period of the perturbations in the boundary layer also changes, which indicates that the frequency of the perturbations in the boundary layer is not a single frequency. In view of this, the FFT method was adopted to convert the perturbations in the time domain into the frequency domain, and to analyze the evolution of perturbations with different modes in the boundary layer. The FFT equation is as follows:

(9) $$p^{\prime }(x,y,t)=Re(\sum_{n=0}^{N}\left|{p^{\prime }}_{n}(x,y)\right|e^{i\left[-nft+\varphi_{n}(x,y)\right]})$$

where *f* represents the frequency of the free-stream disturbances, *p*′(*x*,*y*,*t*) represents any instantaneous pressure disturbance, and $\left|{p^{\prime }}_{n}(x,y)\right|$ and $\varphi_{n}(x,y)$ represent the amplitude and phase angle of the local perturbations, respectively. The integer *n* represents the wave mode of the disturbance field, where *n* = 0 is the average flow field deformation, *n* = 1 represents the fundamental mode, *n* = 2 represents the second harmonic mode, and so on.

Figure 5 shows the spectrum of perturbations in the boundary layer under different free-stream conditions. Free-stream disturbances lead to the formation of perturbations with different modes in the boundary layer. Again, the non-dimensional frequency of the free-stream disturbances is 0.25, and the perturbations with frequencies of 0.25 and 0.50 in the boundary layer are respectively called fundamental mode, second harmonic mode, and so on. The fundamental and second harmonic modes in the boundary layer begin to appear in the nose region under free-stream disturbances, while any harmonic modes with higher frequencies appear at the edge of the nose region or even further downstream. The perturbations in the boundary layer are concentrated in the fundamental and harmonic modes, and the other frequencies account for a small proportion in the boundary layer. The frequency range of the perturbations in the boundary layer caused by fast waves, entropy waves, and vortical waves is wider, in the range 0 < *f* < 5. For perturbations in the boundary layer caused by fast and entropy waves, the higher the frequency in the boundary layer, the later the harmonic mode appears and the earlier it disappears. The spectral maps in the boundary layer caused by fast waves and entropy waves are similar. As the pressure fluctuations in the time domain in the instantaneous hypersonic unsteady flow field under fast waves and entropy waves are similar, we think that the hypersonic boundary layer receptivity under fast waves and entropy waves is similar, whereas the influence of fast waves on the hypersonic flow field and the boundary layer is stronger. For slow waves, the frequency of perturbations in the boundary layer is lower, in the range 0 < *f* < 2.5.

**Figure 6:**
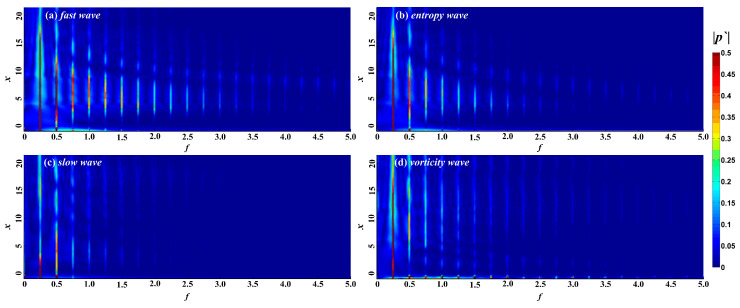
Amplitude diagram of perturbations in the boundary layer under the smooth wall.

**Figure 7:**
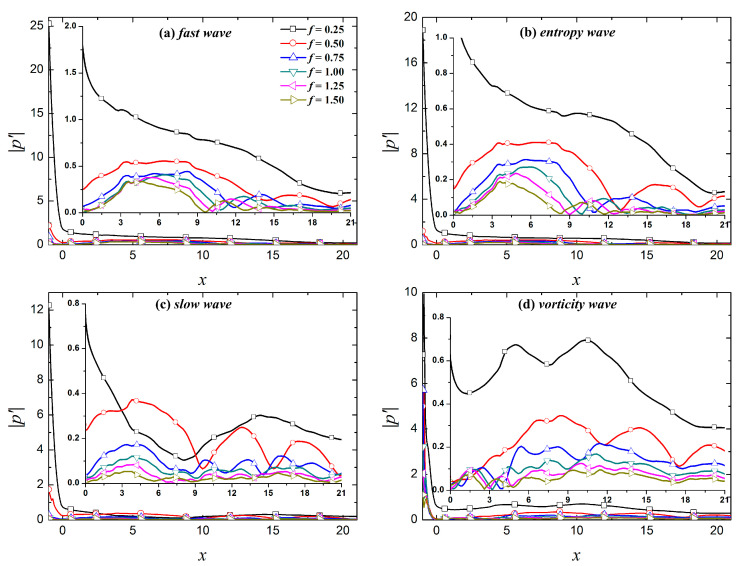
Evolution of perturbations along streamwise under different type free-stream disturbance waves.

Figure 6 and Figure 7 show the evolution of fundamental and harmonic modes in the boundary layer under the free-stream disturbances to provide a clearer depiction of the evolution of different modes in the boundary layer. Figure 6 shows that the dominant modes of the perturbations in the boundary layer caused by fast waves, entropy waves, and vortical waves are all fundamental modes. The fundamental modes caused by fast and entropy waves continuously decrease over the evolution process, whereas the fundamental mode caused by vortical waves demonstrates growth during the evolution process. In the downstream region *x* > 10.37, the fundamental mode continues to decrease. For slow waves, the dominant mode of the perturbations in the boundary layer is not always the fundamental mode. The dominant mode in the boundary layer is the second harmonic mode in the region of 2.95 < *x* < 9.02, and the dominant mode in the rest of the computational domain is the fundamental mode.

**Figure 8:**
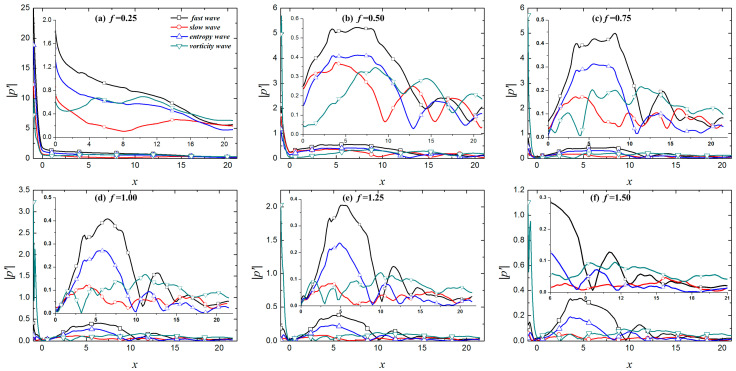
Streamwise evolution of different frequencies of perturbations.

In Figure 8, in the upstream region (*x* < 10) of the calculation domain, the fundamental mode and harmonic modes (except the sixth harmonic mode in the region of 8.90 < *x* < 10.26) in the boundary layer caused by fast waves are stronger than the other three free-stream disturbances. In the downstream region of the calculation domain, the perturbations in the boundary layer caused the vortical waves are larger than the other three disturbances. The amplitude of the perturbations in the boundary layer caused by slow waves is relatively small within the calculation domain, especially for perturbations with *f* > 0.75. In general, the fast waves result in large amplitude fundamental and harmonic modes in the boundary layer in the response phase; their amplitudes gradually decrease in the process of downstream evolution. This is similar to the simulation results reported by Cerminara et al. [37]. They stated that this occurs because the fast waves resonate with the boundary layer when passing through the shock (especially a normal shock), resulting in a significant increase in the amplitude of the perturbations in the boundary layer. The initial amplitude of the perturbations caused by vortical waves is small after entering the boundary layer. As the perturbations in the boundary layer evolve downstream, the amplitude of the fundamental and harmonic modes is gradually larger than the amplitude of the perturbations in the boundary layer under the other three free-stream disturbances. The amplitude of the perturbations in the boundary layer caused by slow waves is relatively small throughout the whole calculation domain because the propagation speed of the perturbations in the boundary layer increases when the perturbations enter the boundary layer. The resonance of the perturbations in the boundary layer caused by slow waves is relatively weak in the nose region, resulting in the initial amplitude of the perturbations in the boundary layer being relatively small. As perturbations evolve downstream, the amplitude of the fundamental and harmonic modes is always small or even the smallest observed.

## 5. Effect of Roughness Element on Boundary Layer Receptivity under Free-Stream Disturbances

**Figure 9:**
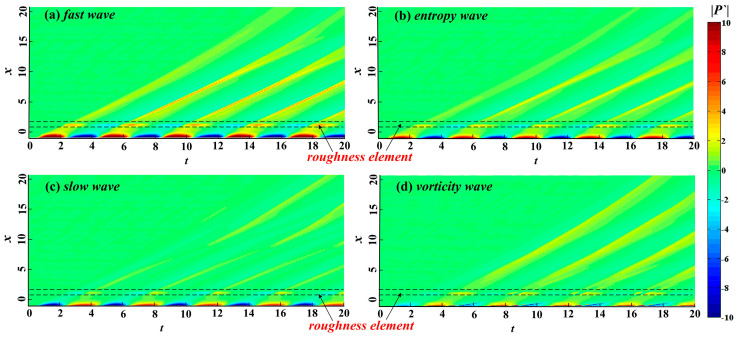
Space-time pressure fluctuation contour map under a wall with a roughness element.

We reported above that free-stream disturbances obviously change the hypersonic boundary layer receptivity, and the evolution processes of the perturbations in the boundary layer under different types of free-stream disturbance are quite different. As another important factor affecting the hypersonic boundary layer receptivity, the wall condition also can affect the propagation of perturbations in the boundary layer. We discussed the impact of a roughness element on the evolution of perturbations in the boundary layer with entropy waves. We depicted the impact of roughness element height on the propagation of perturbations with different frequencies in the boundary layer [24]. Therefore, the response of a hypersonic flow field to free-stream disturbances with a roughness element is not analyzed here. Figure 9 shows the propagation process of wall pressure fluctuations in the spatial and time domains under different types of free-stream disturbance with a rough wall. In the spatial domain, when perturbations in the boundary layer encounter the roughness element, the wall pressure fluctuations are amplified. When the perturbations pass through the center of the element, pressure fluctuations begin to decrease until the downstream region of the roughness element. In the time domain, perturbations in the boundary layer are continuously affected in the roughness element region, and the wall pressure fluctuations are always amplified. Generally, the roughness element has a similar influence on perturbations in the boundary layer with different free-stream disturbances. The only difference is that the variation in the amplitude of the perturbations in the boundary layer has a certain influence. Among the four kinds of free-stream disturbances, the roughness element has the strongest amplification effect on the perturbations in the boundary layer caused by fast waves, followed by entropy waves, and the perturbations in the boundary layer caused by slow and vortical waves are the weakest.

**Figure 10:**
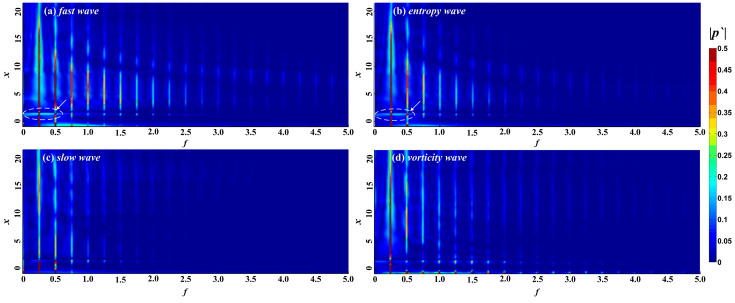
Amplitude diagram of perturbations in the boundary layer under the wall with a roughness element.

**Figure 11:**
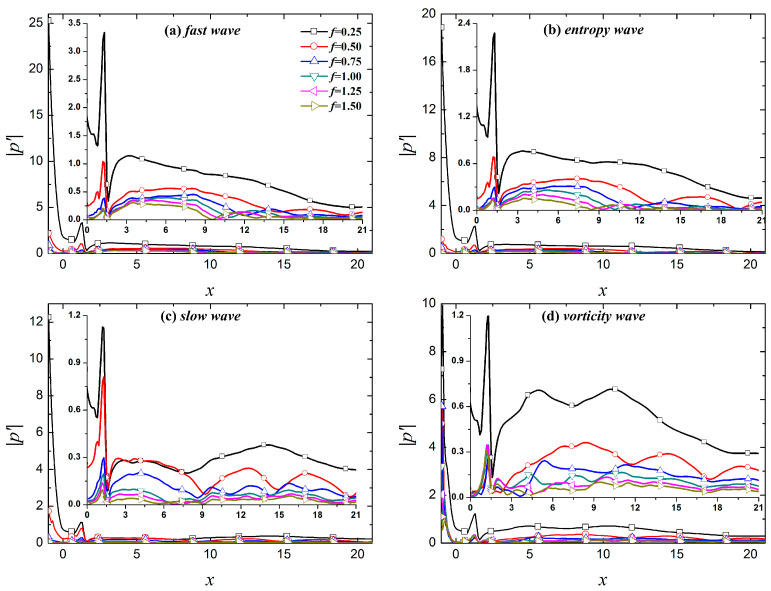
Streamwise evolution of perturbations in the boundary layer along under different types of free-stream disturbance waves.

FFT was adopted to further examine the influence of roughness elements on the propagation of perturbations in the boundary layer under free-stream disturbances. The impact of roughness elements on the propagation of perturbations in different modes in the boundary layer was analyzed. Figure 10 shows the spectral analysis of different modes in the boundary layer under roughness elements. The roughness element has a certain effect on the fundamental mode and harmonic modes. The new perturbations in the boundary layer caused by fast waves and entropy waves are formed in the roughness element region, as shown by arrows in Figure 10. The roughness element results in the amplification of the fundamental mode in the leading edge region of the roughness element and the suppression of the trailing edge region of the roughness element. Figure 11 shows the distribution of fundamental mode, and second, third and fourth harmonic modes along the flow direction in the boundary layer of the roughness element under four kinds of free-stream disturbances, and compares the evolution of the perturbations between the smooth wall and the rough wall. In the figure, "NR" represents the smooth wall and "R" represents a rough surface. The roughness element has a significant impact on the evolution of perturbations for different modes in the boundary layer. In general, the roughness element has the greatest influence on the fundamental mode and harmonic modes in boundary layer under slow waves, followed by vortical waves and entropy waves, and fast waves least influence the perturbations in the boundary layer. For the boundary layer under fast waves, entropy waves, and vortical waves, the influence of the roughness element on the perturbations in the boundary layer was approximated, and the fundamental mode and the harmonic modes were amplified in the upstream half of the roughness element and suppressed in the downstream half region of the roughness element. The fundamental mode is continuously amplified after passing through the roughness element, but the amplification effect is limited. The amplification effect gradually weakens as the fundamental mode evolves downstream, and the fundamental mode is always the dominant mode in the boundary layer. The roughness element also greatly influences the evolution of the perturbations in the boundary layer under slow waves. In addition to the phenomenon where the perturbations in the boundary layer are amplified in the upstream half region of the roughness element and suppressed in the downstream half region of the roughness element, the evolution of perturbations in the boundary layer in the downstream region of the roughness element is continuously influenced by the roughness element. In the downstream region of the roughness element, the fundamental mode is continuously amplified in the region of *x* > 3.54, while the second harmonic mode is suppressed to *x* = 9.41, showing oscillatory propagation in the further downstream region. Notably, the roughness element changes the mode competition mechanism of the perturbations in the boundary layer. The downstream region of the roughness element is mainly dominated by the fundamental mode. Although the second harmonic mode is higher than the fundamental mode in some regions, the amplitudes of the two are similar. This is an essential difference from the modal competition mechanism of perturbations in the boundary layer caused by slow waves between the rough wall and smooth wall. With a rough wall, the dominant mode in the hypersonic boundary layer under free-stream disturbances is always the fundamental mode.

## 6. Conclusions

A high-precision method was adopted to numerically simulate a hypersonic flow field with free-stream disturbances under different wall conditions. The process of continuous free-stream disturbances entering a hypersonic flow field through shock and propagating in the flow field was described. The generation and evolution of perturbations in the boundary layer under free-stream disturbances were analyzed. The propagation of perturbations in the boundary layer under different types of free-stream disturbance in the time and space domains was compared. We analyzed the impact of a roughness element on the propagation of different modes in the boundary layer. We drew the following conclusions:

(1) Free-stream disturbances strongly influence the hypersonic flow field, with acoustic waves most seriously influencing flow field, followed by entropy waves, and vortical waves had the least influence. The influence of slow acoustic waves on the flow field mainly concentrates in the region between the shock and the boundary layer, whereas the influence of fast acoustic waves is mainly concentrated in the boundary layer.

(2) The amplitude of perturbations in the boundary layer is significantly larger in the nose region than in the downstream region. In the upstream half of the computational domain, the amplitude of the perturbations in the boundary layer caused by fast acoustic waves is the largest, whereas the amplitude caused by vortical waves is the largest in the downstream half. The response process of the boundary layer to free-stream disturbances can be divided into a response phase and a periodic phase. The amplitude of the perturbations in the boundary layer during the response and periodic phases is influenced by the location and the type of free-stream disturbances.

(3) Multimodal perturbations form in the boundary layer caused by free-stream disturbances, wherein the fundamental mode is the dominant mode of perturbations in the boundary layer caused by fast acoustic waves, entropy waves, and vortical waves. The dominant modes of perturbation in the boundary layer caused by slow acoustic wave are the fundamental mode and the second harmonic mode. The effects of fast acoustic waves and entropy waves on boundary layer receptivity are similar. The difference is that the amplitude of the perturbations in the boundary layer caused by the fast acoustic waves is larger.

(4) The roughness element changes the evolution process of perturbations with different frequencies in the boundary layer. The fundamental mode and harmonic modes are significantly amplified in the upstream half region of the roughness element and suppressed in the downstream half region of the roughness element. In the downstream region of the roughness element, the perturbations in the boundary layer caused by slow acoustic waves have the greatest influence, the second harmonic in the boundary layer is significantly suppressed, and the fundamental mode becomes the dominant mode. The fundamental mode remains the dominant mode of the perturbations in the boundary layer caused by the fast acoustic waves, entropy waves, and vortical waves, and the fundamental mode is slightly amplified.

## Figures and Tables

**Table 1:** The parameters of free-stream flow.

∞ (°)	*Ma*_∞_	*Re*_∞_	*T*_∞_
0	6	10,000	169

**Table 2:** The geometric parameters of the model.

*R*	*θ*	*h*	*w*	*x_c_*
1	5	0.15	0.40	1.4014
